# Breaking Bad News: The Perspective and Experience of Women with Gynecological Cancer (Results of the NOGGO-Expression XX Survey)

**DOI:** 10.3390/curroncol33040229

**Published:** 2026-04-18

**Authors:** Ela Igde, Gülten Oskay-Özcelik, Jekaterina Vasiljeva, Murat Karaman, Susanne Fechner, Adak Pirmorady Sehouli, Jalid Sehouli

**Affiliations:** 1 Department of Gynecology with Center for Oncological Surgery, Charité—Universitätsmedizin Berlin, Corporate Member of Freie Universität Berlin and Humboldt-Universität zu Berlin, 13353 Berlin, Germany; ela.igde@charite.de; 2 North-Eastern German Society of Gynecological Oncology (NOGGO), 13359 Berlin, Germany; guelten.oskay@gmx.de; 3 Department of Gynecology and Obstetrics, Vivantes Klinikum am Urban, 10967 Berlin, Germany; jekaterina.vasiljeva@vivantes.de; 4 Statistical Analysis Agency, Berlin, Germany; info@myauswertung.de; 5 German Ovarian Cancer Foundation, 13359 Berlin, Germany; sfechner@stiftung-eierstockkrebs.de; 6 Department of Psychosomatic Medicine, Charité—Universitätsmedizin Berlin, Corporate Member of Freie Universität Berlin and Humboldt-Universität zu Berlin, 12203 Berlin, Germany; adak.pirmorady@charite.de

**Keywords:** delivering bad news, patient preference, communication, migration background, gynecologic oncology

## Abstract

**Background:** Effective communication improves patient satisfaction and reduces stress for both patients and physicians. Surveys consistently highlight the importance of strong communication skills among physicians, especially in oncologic settings. Yet, communication training is neither ubiquitous nor standardized in medical studies or residency, and physicians report that this task represents a burden for them. Given the limited data addressing the observations and expectations of patients with gynecologic malignancies when receiving bad news, this survey aimed to assess their perspective on this topic. **Methods:** We examined throughout an anonymous questionnaire how patients with gynecological and breast cancer experienced the delivery of bad news. Data were collected in Germany from July 2024 to September 2025. The questionnaire was available online and in paper form in four languages (German, English, Turkish, Arabic), with the purpose of recording culture-specific data. **Results:** A total of 249 patients completed the survey. Regarding the overall need for improvement in delivering bad news, 222 women (94.5%) declared that improvement was necessary, with 92 (39.1%) of them indicating that substantial improvement was required. While 67.9% of patients were content with the physician’s professional competence, 30.5% stated a lack of empathy, and 32.9% stated insufficient time for conversation. When comparing satisfied and dissatisfied patients, significant differences were observed across several aspects, such as consultation length, nonverbal communication, calmness of the setting, stress level after the conversation, and the offer to bring a trusted person or arrange a follow-up conversation. **Conclusions:** This patient survey highlights a persistent gap between patients’ expectations and physicians’ performance when it comes to delivering bad news. The findings underline the urgent need for the implementation of systematic training programs and structured communication protocols in gynecologic oncology.

## Simple Summary

Delivering bad news is an integral part of professional routine, not only in gynecologic oncology but in many medical disciplines. Despite its frequency and ordinariness, such conversation remains challenging for many physicians. For improving these interactions, it is essential to understand patients’ expectations. This survey explores the experiences and expectations of patients in gynecologic oncological departments when being delivered bad news. Many women appreciated the physician’s medical competence; however, shortcomings in empathy and time for conversation were identified. Patient satisfaction appeared to be associated with a calm setting, appropriate nonverbal communication by the doctor, the opportunity to ask questions, the option to bring a trusted person, and the offer of a follow-up conversation. Further, patients wished for good news to also be communicated whenever it occurs. These findings should be used to optimize communication strategies in gynecologic oncology when conveying bad news to patients.

## 1. Introduction

The delivery of bad news remains a challenging task, even though it is a commonly and frequently required responsibility in gynecologic oncology. Buckman [1] already suggested in 1984 that ongoing doctors should get communication skills training, especially when working in oncologic fields, emphasizing that conveying bad news is an important and daily part of the job. He claimed that communication skills can be trained and learned, the same as surgical skills, and urged for a larger representation of this topic in the curriculum. Statements and recommendations acknowledged by Fallowfield and Jenkins [2] in 1999, affirming that effective communication is an essential skill for providing good cancer care. In this context, Buckman defined bad news as “any information likely to alter drastically a patient’s view of his or her future” [1].

A recent survey on almost 800 doctors and 300 medical students in German-speaking European countries revealed how this task remains a burden for them and confirmed the enduring need for communication training [3]. The survey also showed that only half of the medical students and two-thirds of the surveyed doctors have learned adequate communication skills. An international survey on healthcare providers working in intensive care units, with more than 3000 participating physicians and nearly 3000 medical students from around 40 countries, reported that merely 40.2% of the surveyed physicians and 26.6.% of the surveyed medical students had received formal training in delivering bad news [4]. Evidence suggests that teaching medical students how to deliver bad news improves both their communication skills and confidence when conducting these conversations, such as in the use of the six-step SPIKES protocol, which increases confidence in oncologists and oncology trainees by implementing key items when conveying bad news, hence standardizing and structuring the conversation [5,6]. In recent years, there has been a shift from the traditional concept of breaking bad news toward a broader framework of serious illness communication, which emphasizes ongoing, patient-centered discussions about prognosis, values, and care preferences throughout the disease trajectory [7,8]. These conversations are increasingly recognized as interprofessional, with nurses, social workers, and other healthcare professionals playing a crucial role in facilitating effective communication and supporting both patients and their families [9,10]. Structured communication interventions, such as those within the Serious Illness Care Program, have been shown to enable earlier, more frequent, and more goal-concordant conversations, thereby improving the effectiveness of physician-patient communication [9,11]. Interventions within the Serious Illness Care Program have also been associated with reduced anxiety in patients with advanced cancer [8]. Furthermore, communication skills training is likely to improve patients’ satisfaction as well as physicians’ empathy and to reduce physicians’ burnout [12,13]. Greater patient satisfaction and reduced emotional distress were associated with the attentiveness and empathy of the physicians during the conversation [14]. Correlations between physicians’ communication and patient adherence [15], as well as patients’ motivation for further therapy, were explored in previous studies [16].

To enhance the conversation while conveying bad news and therefore achieve higher patient satisfaction, we additionally need to be aware of patients’ experiences and expectations [17]. In earlier works, delivering bad news without taking into account the patients’ preferences was related to higher psychological distress in these patients [18]. Heterogeneous studies explored the patient’s perspective with varying patient groups, including different types of diseases. A literature review addressing cancer patients’ preferences regarding the communication of bad news reported differences related to sociodemographic and culture-specific factors [19]. Surveys involving cancer patients pointed out significant differences between patients’ preferences and the actual way in which bad news is delivered [20]. Reviews presenting strategies for breaking bad news in gynecologic oncology exist [21], but during our research process, we found only one survey that examined the position of women with gynecological cancer about their perception when receiving bad news exclusively. In the meant study, women with cervical cancer and cervical intraepithelial neoplasia were surveyed, and the authors reported a noticeable disparity between the expectancies of the patients and the reality when receiving bad news, together with a general need for improvement [22]. The aim of the present study was to fill the gap in gynecologic oncology and to examine specifically the perspectives of women with gynecological and breast cancer regarding the delivery of bad news. We hypothesized that patients perceive a need for improvement, and we intended to find more precise information about their preferences and improvable aspects.

## 2. Materials and Methods

This survey was conducted between July 2024 and September 2025 at two gynecologic departments in Berlin, Germany (at *Charité—Universitätsmedizin Berlin, Campus Virchow-Klinikum* and its affiliated academic teaching hospital, *Vivantes Klinikum am Urban*). Women aged 18 years or older and diagnosed with gynecological or breast cancer were eligible to participate, irrespective of the disease stage or treatment phase. Since we aimed to analyze the processes within the German healthcare system, only women living in Germany were included.

### 2.1. The Questionnaire

A two-part, semi-structured questionnaire containing 61 questions was developed. Participants could choose between a paper-based and an online version; the participation was anonymous. The questionnaire was created based on items of earlier NOGGO surveys [3,23], with the Expression surveys specifically designed to capture the patient perspective in gynecologic oncology—including, in part, a focus on individuals with a migration background [23]—while Herzog et al. [3] address the physicians’ perspective on the topic of breaking bad news; additional questions were generated based on the authors’ discussions and ideas following a literature research on this topic. The first part of the questionnaire gathered sociodemographic data as well as information about the oncological disease. The second part included questions about the experiences and observations the women made while being delivered their subjectively worst news, and the expectations they had. Most of the items were single- or multiple-choice questions, with a smaller number of rating scale questions. Some items had an additional free-text option. The questionnaire used in this study is provided as Supplement S1 in the Supplementary Material. A pilot study was conducted on five patients who met the inclusion criteria to determine comprehensibility and feasibility. Initially, the questionnaire was administered in an interview format, and minor revisions were made to improve clarity. Subsequently, patients completed the questionnaire independently, the required time was recorded, and participants provided feedback regarding potential ambiguities or misinterpretations.

We intended to put a special focus on women with a migration background and therefore provided questionnaires in four languages. The online version was available in German, English, and Turkish; the paper-based version was additionally available in Arabic. The questionnaire was originally designed in German; the translation was done using OpenAI’s ChatGPT and was then adjusted and corrected by our native-speaking authors and co-authors. The Arabic version has been directly translated from the German version without using any artificial intelligence support.

On the first two pages of the questionnaire, women were briefed on the aim of the study, the inclusion criteria, and the voluntary nature of participation. By answering the questions anonymously and by submitting the questionnaire, patients gave their consent to participate in the study. The Charité Ethics Committee in Berlin (application number: EA1/024/24) approved the questionnaire as well as the type of informed consent. The survey was coordinated by the North-Eastern German Society for Gynecological Oncology (NOGGO) and supported by the German Ovarian Cancer Foundation (“Deutsche Stiftung Eierstockkrebs”).

### 2.2. Statistical Analysis

For building and managing the online survey, we used Research Electronic Data Capture (REDCap, version 15.0), a browser-based software that also acted as our database. After entering the data from the paper questionnaires into our REDCap database, we exported all the records to SPSS for the statistical analysis. Using SPSS (IBM SPSS Statistics, version 27.0), we first created a report of all questions by frequency. We recorded missing responses for each item of the questionnaire, marked as ‘N/A’ (not available) in the tables, and we did not include them in the analysis. For nominal and ordinal data, reports with absolute numbers and percentages were created. For ratio data, we reported the median, mean, minimum, and maximum values, as well as the standard deviation. Descriptive statistical analysis was applied to visualize the patients’ characteristics. We conducted exploratory subgroup analyses using the Mann-Whitney U test for nonparametric data and Chi-Square tests for categorical data. The sample size of this survey was based on the statistical precision to estimate the primary endpoint, with a maximum variance set at 50%. A *p*-value < 0.05 was considered statistically significant. The results of the significance tests for the statistical analysis of the subgroups are exploratory, not confirmatory. Further, we performed univariable and multivariable logistic regression analysis to examine associations between patients’ characteristics and their perceptions in the breaking bad news conversation and to detect potential confounders.

## 3. Results

A total of 281 women participated in the survey by submitting a questionnaire. Twenty-four participants were excluded due to incomplete answering, five did not have a gynecologic oncological disease (instead specifying conditions such as endometriosis or other types of cancer), and three did not specify their diagnosis. The final analysis included a total of 249 participants. Table 1 provides information about the participants’ sociodemographic data, their diagnoses, and the worst news they received in the course of their illness.

When asked about the overall need for improvement in delivering bad news, 222 women (94.5%) declared a need for improvement, with 92 women (39.1%) indicating that much improvement was required. One hundred and sixty-nine (67.9%) patients stated being satisfied with the doctor’s professional competence, whereas only 95 (38.2%) stated being satisfied with the doctor’s empathy, and 87 (34.9%) with the doctor’s motivational attitude. In addition, 223 (93.7%) respondents indicated that they would like to receive good news more frequently.

**Figure 1:**
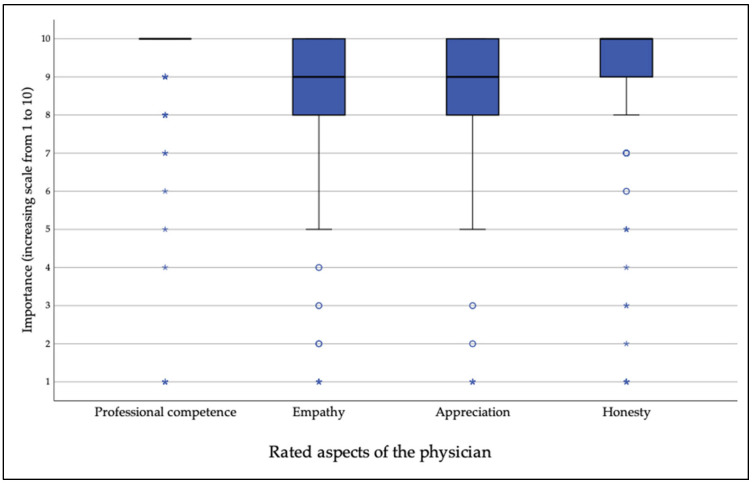
Different aspects of the physician, ranked by importance from 1 to 10. ○ moderate outliers * extreme outliers.

Figure 1 represents how patients rated different aspects of physicians on a scale from one to ten, depending on how important each aspect was to them. Professional competence was ranked highest (9.44), followed by honesty (9.22), appreciation (8.55), and empathy (8.51).

Women were also invited to specify their level of satisfaction concerning the conversation in which they were given the bad news; the response options were ordered on a 5-point Likert scale varying from *very satisfied* to *very dissatisfied*. Fifty-eight patients stated, respectively, to be *very or rather satisfied* (each 23.5%), 52 patients chose the option *neither nor* (21.1%), 37 answered *rather dissatisfied* (15.0%), and 42 answered *very dissatisfied* (17.0%). Two participants did not answer this question. Using this item, we grouped the answers and created two subgroups: patients who were satisfied (*n* = 116, 47.0%) and dissatisfied (*n* = 79, 32.0%) with the conversation. The “neither satisfied nor dissatisfied” category was excluded from this analysis. Table 1 shows additional patient characteristics of these two subgroups, named “satisfied patients” and “dissatisfied patients”, as well as their comparison.

When analyzing patient characteristics of the two subgroups, we determined a difference concerning the patients’ age, the dissatisfied group being statistically significantly younger than the satisfied group (*p* = 0.014). For other sociodemographic factors, we did not determine a significant difference between the groups when examined individually. We additionally performed a multivariable logistic regression using six variables of the patient’s characteristics, examining their relation to satisfaction; the results are shown in Table 2. When looking at the chosen variables in a multivariable regression model, we can see an association between higher age and greater satisfaction. In addition, women with a migration background appear to be rather satisfied with the conversation, and women with a university degree appear to be rather dissatisfied with the conversation. However, the effect of the migration background on satisfaction is different when adjusting for patients’ age (OR = 1.948, 95%-CI: 0.928–4.089; *p* = 0.078 vs. OR = 2430, 95%-CI: 1110–5321, *p* = 0.026); therefore, the age is a confounder in this relation.

Table 3 contains items of the second part of the questionnaire, addressing patients’ experiences and expectations, and shows the comparison of the two named subgroups. In addition to the items in Table 3, we found that 50% of the patients who were dissatisfied with the conversation were not offered to bring a trusted person, and only 18.4% of patients in this subgroup got such an offer. Further, 31.6% indicated that the conversation took place without prior appointment, compared to 57.1% of the satisfied group, who reported having been offered to bring a trusted person (X^2^(2) = 28.255, *p* < 0.001). Also, the dissatisfied patients group felt rather overwhelmed when communicating the bad news to their trusted person (X^2^(4) = 19.220, *p* = 0.001) and were more likely to wish for support while conveying the bad news to their relatives (X^2^(2) = 8.556, *p* = 0.014).

**Figure 2:**
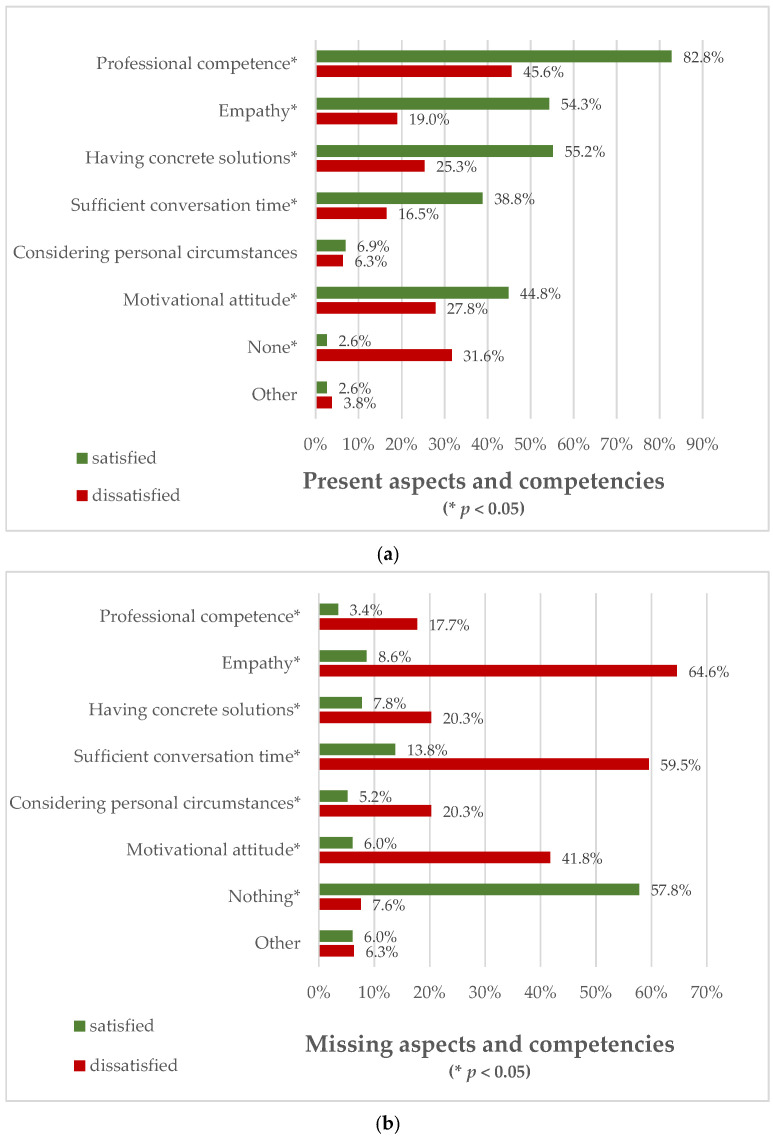
Aspects and competencies of the physician rated by the surveyed patients, being present and satisfactory (**a**) or missing (**b**); patients could give multiple answers for these items.

Figure 2 illustrates aspects and physicians’ competencies that were present or missing during the conversation for each subgroup. When asked about improvable factors, 43% of the participants reported that they would have wished for more time for the consultation.

When patients were asked about the effect of the conversation on their relationship with the physician, 65.2% patients of the satisfied group reported that the relationship was greatly or rather strengthened, while only 2.7% indicated that it was rather weakened, and no one reported that it was greatly weakened. Whereas 32.4% of the dissatisfied patients answered that the relationship was greatly weakened, 23.0% answered that it was rather weakened, and only 14.9% answered that it was greatly or rather strengthened.

As we wanted to put a special focus on women with a migration background, we defined two groups: first- and second-generation migrants, according to the definitions of the European Migration Network (EMN) glossary [24]. In the group of first-generation migrants, we placed women who were not born in Germany and whose parents were not born in Germany. The second-generation group included women who were born in Germany, but whose parent(s) (at least one) were not born in Germany. Thirty-eight (15.3%) patients met the criteria for the first-generation migrant group, and 18 (7.2%) patients for the second-generation migrant group. When summed up, we had one group of 56 (22.5%) women with a migration background, defined as persons who themselves or whose ancestors immigrated to Germany [25]. Women with a migration background were significantly younger than those without a migration background (mean age of 56 years and 60 years, respectively, *p* = 0.017). The comparison between women with and without a migration background did not determine a significant difference concerning the overall need for improvement (X^2^(3) = 7.571, *p* = 0.056). When comparing first- and second-generation migrants, we could not see any difference regarding the general satisfaction with the conversation (Fisher’s Exact Test, *p* = 0.715). The generational groups were too small to get a valid outcome concerning the need for improvement.

Another subgroup analysis was performed comparing women with ovarian, fallopian tube, and peritoneal cancer (*n* = 185, 74.3%) to other cancer types (*n* = 64, 25.7%). We did not determine either a significant difference concerning the overall need for improvement (X^2^(3) = 0.106, *p* = 0.991) or the general satisfaction with the conversation (X^2^(4) = 4.236, *p* = 0.375). Furthermore, we did not find other items with statistically significant results while comparing these two subgroups.

## 4. Discussion

The findings support our hypothesis, with 95.4% of the surveyed patients responding that the delivery of bad news should be improved. The proportion of satisfied patients in our survey (47.0%) was similar to some previous research, which reported 46.2% of cancer patients being satisfied with the conversation when receiving bad news [20] or 47% of the patients indicating “that the doctor delivered the bad news in a proper way” [16]. However, our results differ from the recent survey on women with cervical cancer and intraepithelial neoplasia (CIN), where 72% of the patients specified to be satisfied with the way they received bad news; though, the number of satisfied women was highest in the group of patients with CIN and considerably less in the groups of women with cervical cancer [22].

Patients consistently identified professional competence as the most important competency of the doctor. With two-thirds of the patients (67.9%) being satisfied with it, professional competence was evaluated as the most satisfactory item in our study, in agreement with an earlier survey on expectations of breast cancer patients, ranking highest the physician’s competence [26]. This was similar to the findings of other studies, in which cancer patients ranked the item that doctors should be informed about the recent cancer research notably high when surveyed on their preferences regarding the delivery of bad news [27,28]. Patients are generally not asked about their expectations concerning good news. Our analysis showed that patients in the satisfied group were more likely to have received any good news. When considering the entire cohort, nearly all participants (93.7%) stated that physicians should communicate good news more frequently.

Many participants specified in recurrent items the need for more time during these conversations, in line with previous studies on breaking bad news [22,29], apparently remaining a big obstacle in many healthcare systems. Another component that patients expected regarding the general setting of the conversation was a calm and undisturbed environment, which has also been documented in many prior surveys [22,29,30] and seems to contribute to a higher satisfaction level.

Patients should be offered the opportunity to be accompanied by a relative during the conversation. Previous research highlighted that this decision is highly individual; the preference for being accompanied or attending alone depended strongly on the surveyed population [31,32]. For instance, a literature review on cancer patients’ preferences elaborated that Asian patients preferred to have their relatives with them when receiving bad news more than Westerners [19]. Nevertheless, patients expressed a clear preference for having the option to bring a relative to such conversations, a finding that is consistent with surveys of bad news communication in specialties outside gynecologic oncology [33]. Physicians reported at 57.4% to encourage patients to be accompanied by their relatives [3], reflecting an existing deficiency concerning this offer.

Another offer that participants expect is a follow-up conversation, an observation supported by other surveys about bad news disclosure [34,35]. Patients only partly remember the information they get during a conversation with their physician, especially when getting a cancer diagnosis [36,37]. The recall of information appears to be lower when the information given causes anxiety or physical arousal, both likely to happen when receiving bad news [38,39,40]. Therefore, patients request a follow-up appointment as well as written information [34] or suggestions for finding valid information about their diseases, along with recommendations for psychological support, such as support groups [35,41].

Overall, women who were dissatisfied with the conversation presented a higher stress level and appeared to experience greater difficulties when communicating the bad news to their relatives. Moreover, they stated to have a weakened relationship to their treating physician, demonstrating that a physician who performs poorly while delivering bad news may have negative implications that go beyond the conversation itself and may even affect the patients’ further treatment, as earlier studies report [15,16]. Particularly little is known about cancer patients conveying the bad news they have received to their relatives. Previous surveys indicated that several cancer patients face great difficulties in these situations without elaborating on details [31]. This topic should be considered for further research. This is also an aspect that highlights the importance of nurses and social workers, who are closely involved in daily patient care and in communicating different dimensions and aspects of care to family members, underscoring the need for their active involvement and education, as well as for a multidisciplinary approach in general [9,10,42].

Previous studies have shown that patients feel helpless when receiving bad news by phone [41] and prefer to be informed in person [27,28]. In our survey, the proportion of women receiving bad news over the phone or through other means of communication was higher among dissatisfied patients. This is a fact that should be taken into account, especially when considering the results of a survey of breast cancer patients in the United States, indicating that the number of patients receiving their cancer diagnosis over the phone has increased significantly over the last twenty years [43].

We sought to identify sociodemographic factors associated with higher satisfaction; however, age emerged as the only statistically significant factor in this relation, with a higher mean age observed among satisfied participants. Our multivariable regression analysis showed that younger patients with a higher academic background are rather dissatisfied with the conversation and that women with a migration background are, contrary to our expectations, rather satisfied, especially despite the fact that participants with a migration background were significantly younger than participants without a migration background. Recent surveys, even if not carried out on cancer patients only, concluded that younger women have higher expectations when being delivered bad news [16]. This also appeared in a systematic literature review about cancer patients’ preferences regarding bad news communication, seeing an association between female, younger, and more highly educated patients, respectively, and the wish to receive as much detailed information as possible [19]. The Expression III survey examined the preferences of women with ovarian cancer regarding the physician-patient relationship and, likewise, observed higher information needs in younger patients [44]. It seems that younger women with a higher academic background have greater expectations and wish for more precise consultations, requirements that the physicians apparently do not fulfill sufficiently. Concerning the higher satisfaction of women with a migration background, one may hypothesize that these women have lower expectations that may stem from experiences they had in their country of origin, with an eventually more paternalistic physician-patient relationship or poorer physician-patient communication. This would at least be a possible explanation for the group of first-generation migrants and would also explain the higher satisfaction despite the younger age. One would therefore expect that second-generation migrants would be dissatisfied, similar to women without a migration background, since they would have grown up in the same (medical) system; however, our subgroup analysis did not determine a difference in terms of satisfaction between first- and second-generation migrants. Likewise, the Expression V survey conducted among women with ovarian and breast cancer showed no differences between participants with and without a migration background when evaluating the satisfaction concerning the physician-patient communication [23], therefore making it difficult to generalize and indicating the need for further research.

### 4.1. Limitations

In our study, the group of dissatisfied patients was significantly younger than the group of satisfied patients and may therefore have higher expectations, as discussed above. Having two groups that are homogeneous in terms of sociodemographic factors would improve the validity of the results.

Our survey had an overrepresentation of women with ovarian, fallopian tube, and peritoneal cancer since data were mainly collected at a center specializing in these malignancies. Even if our subgroup analysis didn’t reveal significant differences between these patients and patients with other cancer types, one might expect differences related to distinct disease progressions.

Considering that patients could participate in our survey regardless of where they were in the disease course, our study group was quite heterogeneous, including, for example, women at initial diagnosis, during chemotherapy, at cancer recurrence, or in follow-up care. Therefore, time after their initial diagnosis is varying, and their perception may have changed over time. We did not make a difference between women with primary and recurrent disease. The actual timing of the assessment and the patient’s condition at that time may also influence the perception and, consequently, the way of answering the survey. Moreover, patients were invited to fill out the questionnaire considering the conversation with the worst news that they were given, which is highly individual and greatly differing in dependence on the illness course. However, for most of our participants, the worst news received was the confirmed or suspected diagnosis. This finding is consistent with another survey in which cancer patients reported that receiving the diagnosis was the subjectively worst news, regardless of cancer stage [18].

The cross-sectional design of the study additionally did not allow for seeing if and how the patient perception changed over time. It also did not allow for assessing how communication and care evolve over time, which is not consistent with an ongoing, longitudinal process as envisaged in the newer concept of serious illness communication [7]. Also, we could not establish causalities and could only report the observed associations. The self-report questionnaire that we developed for this survey was not validated.

Despite providing questionnaires in different languages to try to include as many patients with a migration background as possible, our migrant groups, especially when divided into first- and second-generation migrants, remained quite small and not sufficiently representative. Other Expression surveys showed different expectations of patients with a migration background and gynecologic malignancy, concerning, for example, advance directives or information needs in their native language [45]. With the goal of performing better subgroup analyses, our survey is still open for participation by aiming for a bigger population size, especially with a better representation of people with a migration background.

### 4.2. Suggestions

Based on our results, as well as checklists issued from the book by Sehouli [42], we created a ten-point plan of factors that should be considered while breaking bad news to women with gynecological cancer, with professional competence being an unapproachable requirement:

1. Plan enough time for the conversation
2. Offer that the patient bring a trusted person
3. Provide a calm setting without disruption
4. Mind nonverbal communication and empathy
5. Gather the knowledge level of the patient
6. Make breaks and give the possibility to ask questions
7. Avoid giving too much information at once and focus on the key message
8. Provide written informational material or give suggestions on where to find verified information
9. Make an offer for a follow-up appointment and give recommendations for support options
10. Conclude and point out positive aspects

We suggest an analogical consultation checklist for patients [42], as interventions indicate that patient coaching improves patient-centered communication [11]:

1. Prepare for the conversation
2. Consider what benefits you and how you can be supported
3. Decide if you want to be alone or accompanied
4. Think about the amount of information you would like to have; you may ask your trusted person to take notes
5. Express your emotions
6. Mirror what you understand
7. Formulate questions
8. Ask for a break if needed
9. Concentrate on the key message
10. Seek the next steps and ask for a follow-up appointment

A prospective study on women with gynecological cancer could be considered by using these checklists, exploring whether women are more satisfied with the conversation when complying with the items on these checklists.

## 5. Conclusions

To our knowledge, this is the first study to survey a heterogeneous group of women with gynecological and breast cancer regarding their perspectives and experiences on the delivery of bad news, revealing a substantial disparity between their expectations and their subjective perception of the physicians’ performance. This disparity appears to be multifactorial in origin and exists independently of the migration background or the type of gynecological malignancy of the participants. The findings suggest a lack of physicians’ communication skills, time for consultation, and standardized procedures that respond to the patients’ preferences, therefore revealing the urgency for systematic training programs, as well as structured communication protocols in gynecologic oncology.

## Figures and Tables

**Table 1:** Patient characteristics, whole cohort (*N* = 249), and two subgroups.

Patient Characteristics	Total, *n* (%) *N* = 249 (100%)	Satisfied Patients, *n* (%) *N* = 116 (47%)	Dissatisfied Patients, *n* (%) *N* = 79 (32%)	*p*-Value (* Significant)
**Age** (in years, mean ± SD)	59 ± 12	61 ± 12	57 ± 11	0.014 *
N/A	2	0	2	
**Age at initial diagnosis** (in years, mean ± SD)	55 ± 13	57 ± 13	52 ± 11	0.006 *
N/A	1	1	0	
**Marital status**				
Single	33 (13.3)	14 (12.1)	9 (11.4)	0.959
Married	143 (57.4)	70 (60.3)	49 (62.0)	
In a partnership	21 (8.4)	10 (8.6)	7 (8.9)	
Widowed	20 (8.0)	9 (7.8)	4 (5.1)	
Separated/Divorced	32 (12.9)	13 (11.2)	10 (12.7)	
N/A	0	0	0	
**Having child (ren)**				
Yes	180 (72.3)	82 (70.7)	58 (73.4)	0.678
No	69 (27.7)	34 (29.3)	21 (26.6)	
Number of children (mean ± SD)	1.80 ± 0.81	1.78 ± 0.83	1.84 ± 0.76	0.490
N/A	0	0	0	
**Highest educational level**				
No degree, primary school certificate	10 (4.0)	4 (3.4)	1 (1.3)	0.155
Middle/intermediate school certificate	43 (17.3)	19 (16.4)	14 (17.7)	
Technical school diploma	32 (12.9)	18 (15.5)	9 (11.4)	
High school diploma	32 (12.9)	20 (17.2)	6 (7.6)	
University/college degree or higher	131 (52.8)	55 (47.4)	49 (62.0)	
N/A	1	0	0	
**Employment status before diagnosis**				
Employee	148 (59.4)	66 (56.9)	53 (67.1)	not applicable
Self-employment/Freelance	44 (17.7)	19 (16.4)	14 (17.7)	
Student/Trainee	3 (1.2)	1 (0.9)	2 (2.5)	
Unemployed/Unable to work/Temporary leave	9 (3.6)	4 (3.4)	4 (5.0)	
Retired	45 (18.1)	26 (22.4)	6 (7.6)	
N/A	0	0	0	
**Native language**				
German	215 (86.7)	97 (84.3)	72 (91.1)	not applicable
Bilingual	27 (10.9)	13 (11.3)	7 (8.9)	
Language other than German	6 (2.4)	5 (4.3)	0	
N/A	1	1	0	
**Own rating of German language skills**				
Very good	202 (83.1)	88 (77.2)	67 (89.3)	not applicable
Good	25 (10.3)	14 (12.3)	6 (8.0)	
Average	5 (2.1)	2 (1.8)	1 (1.3)	
Poor	8 (3.3)	7 (6.1)	1 (1.3)	
Very poor	3 (1.2)	3 (2.6)	0	
N/A	6	2	4	
**Country of birth**				
Germany	209 (83.9)	93 (80.2)	71 (89.9)	0.069
Country other than Germany	40 (16.1)	23 (19.8)	8 (10.1)	
N/A	0	0	0	
**Parents born in Germany**				
Yes (both parents born in Germany)	193 (77.5)	84 (72.4)	67 (84.8)	0.108
No (at least one parent not born in Germany)	56 (22.5)	31 (26.7)	12 (15.2)	
N/A	0	0	0	
**Gynecological disease**				
Ovarian, fallopian tube, peritoneal cancer	185 (74.3)	91 (78.4)	55 (69.6)	0.163
Endometrial cancer	26 (10.4)	12 (10.3)	9 (11.4)	0.817
Cervical cancer	22 (8.8)	8 (6.9)	7 (8.9)	0.613
Breast cancer	16 (6.4)	8 (6.9)	6 (7.5)	0.853
Borderline ovarian tumor	9 (3.6)	3 (2.6)	2 (2.5)	0.981
Other type of gynecological caner	22 (8.8)	9 (7.8)	8 (10.1)	0.565
More than one gynecological cancer	29 (15.7)	14 (12.1)	8 (10.1)	0.674
N/A	0	0	0	
**Worst received news**				
Suspected diagnosis	40 (16.2)	16 (13.9)	10 (12.8)	0.555
Confirmed diagnosis	109 (44.1)	55 (47.8)	33 (42.3)	
Recurrence	43 (17.4)	20 (17.4)	13 (16.7)	
Disease progression	13 (5.3)	7 (6.1)	4 (5.1)	
Metastasis	25 (10.1)	13 (11.3)	10 (12.8)	
Others (lack of therapy options, therapy failure, side effects of treatment, complications, etc.)	17 (6.9)	4 (3.4)	8 (10.3)	
N/A	2	1	1	

* significant.

**Table 2:** Multivariable logistic regression model assessing the relation between patients’ characteristics and satisfaction with the conversation.

Variable	Coefficient	Wald-X^2^	OR (95%-CI)	*p*-Value
**Age**	0.046	9.203	1.047 (1.017–1.079)	0.002 *
**Being married**	−0.256	0.582	0.774 (0.401–1.495)	0.446
**Having children**	−0.526	1.905	0.591 (0.280–1.247)	0.168
**University degree**	−0.699	4.861	0.497 (0.267–0.925)	0.027 *
**Migration background**	1.156	7.159	3.176 (1.362–7.407)	0.007 *
**Ovarian cancer**	0.382	1.195	1.465 (0.739–2.903)	0.274

X^2^ chi-square, OR odds ratio, CI confidence interval, * significant.

**Table 3:** Questions about the conversation when receiving bad news and answers of the two subgroups (*N* = 195), containing single-choice questions and rating-scale questions.

Addressed Item	Total of Two Subgroups, *n* (%) *N* = 195 (79%)	Satisfied Patients, *n* (%) *N* = 116 (47%)	Dissatisfied Patients, *n* (%) *N* = 79 (32%)	*p*-Value (* Significant)
**Conversation length** (in minutes, mean ± SD)	20.2 ± 14.2	26.3 ± 13.7	11.6 ± 9.9	<0.001 *
N/A	16	11	5	
**Quiet setting**				
Yes	135 (70.3)	99 (86.8)	36 (46.2)	<0.001 *
Partly	35 (11.5)	15 (13.2)	20 (25.6)	
No	22 (18.2)	0	22 (28.2)	
N/A	3	2	1	
**Physician taking enough time**				
Yes	139 (72.8)	111 (96.5)	28 (36.8)	<0.001 *
No	52 (27.2)	4 (3.5)	48 (63.2)	
N/A	4	1	3	
**Physician under time pressure**				
Yes	73 (37.6)	27 (23.3)	46 (59.0)	<0.001 *
No	121 (62.4)	89 (76.7)	32 (41.0)	
N/A	1	0	1	
**Enough time to ask questions**				
Yes	105 (54.7)	87 (75.7)	18 (23.4)	<0.001 *
Partly	60 (31.3)	26 (22.6)	34 (44.2)	
No	27 (14.1)	2 (1.7)	25 (32.5)	
N/A	3	1	2	
**Questions sufficiently answered**				
Yes	105 (55.9)	89 (78.8)	16 (21.3)	<0.001 *
Partly	58 (30.9)	22 (19.5)	36 (48.0)	
No	25 (13.3)	2 (1.8)	23 (30.7)	
N/A	7	3	4	
**Met at level of understanding**				
Yes	152 (78.8)	106 (91.4)	46 (59.7)	<0.001 *
Partly	30 (15.5)	9 (7.8)	21(27.3)	
No	11 (5.7)	1 (0.9)	10 (13.0)	
N/A	2	0	2	
**Satisfied with physician’s non-verbal communication**				
Yes	121 (64.3)	100 (90.1)	21 (27.2)	<0.001 *
Neither nor	27 (14.4)	11 (9.9)	16 (20.8)	
No	40 (21.3)	0	40 (52.0)	
N/A	7	5	2	
**Physician well prepared**				
Yes	62 (32.0)	91 (79.1)	16 (20.3)	<0.001 *
Partly	107 (55.2)	23 (20.0)	39 (49.4)	
No	25 (12.9)	1 (0.9)	24 (30.4)	
N/A	1	1	0	
**Received any good news**				
Yes	162 (83.5)	102 (87.9)	60 (76.9)	0.043 *
No	32 (16.5)	14 (12.1)	18 (23.1)	
N/A	1	0	1	
**Satisfied with amount of information**				
Yes	121 (63.0)	99 (87.6)	22 (27.8)	<0.001 *
Partly	21 (10.9)	8 (7.1)	13 (16.5)	
No	50 (26.0)	6 (5.3)	44 (55.7)	
N/A	3	3	0	
**Remembering the shared information afterwards**				
Yes	99 (51.0)	66 (57.4)	33 (41.8)	0.010 *
Partly	85 (43.8)	47 (40.9)	38 (48.1)	
No	10 (5.2)	2 (1.7)	8 (10.1)	
N/A	1	1	0	
**Offered a follow-up conversation**				
Yes, I took advantage of it	71 (38.2)	49 (44.1)	22 (29.3)	<0.001 *
Yes, but I had no need	26 (14.0)	23 (20.7)	3 (4.0)	
No, but I had no need	41 (22.0)	28 (25.2)	13 (17.3)	
No, I would have liked to have one	48 (25.8)	11 (9.9)	37 (49.3)	
N/A	9	5	4	
**Received bad news by other means of communication (e.g., phone, (e-)mail)**				
Yes	59 (30.3)	25 (21.6)	34 (43.0)	0.001 *
No	136 (69.7)	91 (78.4)	45 (57.0)	
N/A	0	0	0	
**Stress level after the conversation** (increasing 1 to 10 scale, mean ± SD)	7.63 (2.47)	6.87 (2.57)	8.78 (1.80)	<0.001 *
N/A	2	0	2	

* significant.

## Data Availability

The raw data supporting the conclusions of this article will be made available by the authors on request.

## Supplementary Materials

The following supporting information can be downloaded at: https://www.mdpi.com/article/10.3390/curroncol33040229/s1, Supplement S1: English version of the questionnaire.

## Abbreviations

The following abbreviations are used in this manuscript:

NOGGO	North-Eastern German Society of Gynecological Oncology
EMN	European Migration Network
OR	Odds ratio
CI	Confidence interval
X^2^	Chi-Square
N/A	Not Available
CIN	Cervical intraepithelial neoplasia
