# Supplementary material for: Breaking Bad News: The Perspective and Experience of Women with Gynecological Cancer (Results of the NOGGO-Expression XX Survey)
Source: Curr Oncol. 2026 Apr 18;33(4):229. doi: 10.3390/curroncol33040229 (PMC13114406; doi:10.3390/curroncol33040229)
Supplement: Supplementary file 1 [file curroncol-33-00229-s001.zip › curroncol-4193637-supplementary.pdf]

Expression XX

Breaking Bad News: the patient’s perspective

CHARITÉ

CharitéCentrum für Frauen-, Kinder- und Jugendmedizin mit Perinatalzentrum und Humangenetik

Charité | Frauenklinik Campus Virchow-Klinikum | 13344 Berlin

Klinik für Gynäkologie mit
Zentrum für onkologische Chirurgie
Direktor: Prof. Dr. med. Jalid Sehouli

Sekretariat
Tel. +49 (0)30 / 450 564 002
Fax +49 (0)30 / 450 564 900
E-Mail: jalid.sehouli@charite.de

Dear Women,

we would like to invite you to take part in our survey “Breaking Bad News: the patient’s perspective” in the field of gynecological cancer.

Previous surveys have highlighted that doctors often feel poorly prepared or overwhelmed when it comes to delivering bad news. Increasing attention is being paid to the topic of "Breaking Bad News," and medical students are expected to engage with such situations during their studies. However, an essential factor in learning and acquiring communication skills is knowing what our counterparts expect from us. Research on patients' perspectives regarding the delivery of bad news is very sparse, especially the perspectives of women with gynecological cancers. Through this survey, we aim to gain this knowledge to both assess our current standing and generate suggestions for improvement in the future.

Participation requirements include being at least 18 years old and having a diagnosis of a gynecological cancer or cancer of the female reproductive system (breast cancer, uterine cancer, cervical cancer, ovarian cancer, etc.); this diagnosis may be in the past and does not need to be current or currently being treated.

Only women living in Germany will be included. Women who were in Germany only for the medical treatment of their gynecological cancer should not be included, as we aim to study the processes within the German healthcare system with this survey.

This survey is coordinated by the study group of NOGGO (*Nord-Ostdeutsche Gesellschaft für Gynäkologische Onkologie* - North-East German Society for Gynecological Oncology) and is conducted nationwide in hospitals and clinics where patients with gynecological cancers are treated.

The participation in this survey is completely voluntary. Non-participation does not result in any disadvantages. By completing the questionnaire or answering the questions, you agree to participate in the survey. You also confirm that you fulfil the participation requirements, understand the objectives of this survey, and are fully informed about the survey.

This study is conducted anonymously and does not require you to give your name, address, or date of birth. We have tried to limit the number of questions to try to save your time and minimize the effort required to filling it out. Completing the questionnaire will take approximately 30 minutes to 1 hour of your time. Please place the completed questionnaire in the enclosed envelope and return it sealed to the person from whom you received the questionnaire or send it anonymously to the address below.

As an alternative to the paper questionnaire, you can also fill it out online. You can find it at the following link: *https://redcap.charite.de/survey/surveys/?s=8TFH8FC7P8LKMCLN*

You can also scan the QR code to open the online version of the survey.

By submitting the questionnaire, you agree that your responses to the questions can be processed for the above-mentioned survey. The data will not be transmitted to any other parties. The questionnaires will be kept by NOGGO for 10 years and then be destroyed. Please note that the results of the survey may be published in medical literature, but your identity will remain anonymous.

We sincerely thank you for your cooperation and significant contribution to optimizing the conversations and processes for delivering bad news!

Prof. Dr. med. Jalid Sehouli
Study director (Studienleiter)
Director of the Clinic for Gynecology with Center for Oncological Surgery
Campus Virchow Klinikum – Clinic for Gynecology with Center for Oncological Surgery
Augustenburger Platz 1
13353 Berlin

Ela Igde
Physician performing the study
Kontakt: ela.igde@charite.de

Sociodemographic data and migration

1. **How old are you?** _____ years

2. **What is your marital status?**
   - Single
   - Married
   - In a partnership
   - Widowed
   - Separated/Divorced
   - Other: ______________

3. **Do you have children?**
   - Yes,
     How many? _____
   - No

4. **What is your highest education level according to German educational system?**
   - No degree
   - Primary school certificate
   - Middle/intermediate school certificate
   - Technical school/vocational school diploma
   - High School diploma/ advanced technical college certificate (Abitur)
   - University/college degree or higher

5. **What is your current employment status?**
   - Employed
   - Self-employed or freelance
   - Student/pupil/trainee
   - Job seeker/unemployed
   - Unable to work
   - Temporary leave e.g., parental leave
   - Retired

6. **What was your employment status before the diagnosis?**
   - Employed
   - Self-employed or freelance
   - Student/pupil/trainee
   - Job seeker/unemployed
   - Unable to work
   - Temporary leave e.g., parental leave
   - Retired

7. What is the language in which you can best express yourself (i.e. the language you dream in)?

- German
- Other language: _______________
- Bilingual: ________________ and _________________

Which other languages do you speak? _______________________________________

8. How would you rate your German language skills?

- Very good
- Good
- Average
- Poor
- Very poor

9. Where were you born?

- Germany
- Other, please specify _______________________

10. How long have you mainly lived in Germany?

- Since birth
- Less than 6 months
- Between 6 and 12 months
- More than 12 months
- I do not live in Germany

11. Where were your parents born?

Mother:
- Germany
- Other, please specify ______________________

Father
- Germany
- Other, please specify ______________________

12. How long have your parents mainly lived in Germany?

- Mother
  - Since birth
  - Less than 5 years
  - More than 5 years
  - Not residing in Germany
- Father
  - Since birth
  - Less than 5 years
  - More than 5 years
  - Not residing in Germany

13. Which of the following medical conditions applies to you?

- o Breast cancer
- o Uterine cancer
- o Cervical cancer
- o Ovarian cancer
- o Borderline-Tumor of the ovary
- o Other gynecological cancer:
  Please specify: ______________

14. In which year was the inital diagnosis made, i.e. when was the disease first detected?

____

How old were you at that time? _______ years

15. What was the worst news you received in the context of your illness?

- o Suspected diagnosis/suspicion of illness
- o Confirmed diagnosis/confirmation of illness
- o Recurrence (reappearance)
- o Disease progression
- o Metastasis (spread)
- o Lack of therapy options
- o Therapy failure
- o Side effects of treatment, complications
- o Recommendation for surgery
- o Recommendation for chemotherapy
- o Recommendation for radiation therapy
- o Recommendation for hormone therapy
- o Recommendation to participate in a study

The following questions refer to the conversation with medical staff in which <u>the worst news</u> (according to you) was communicated to you.

16. Were you satisfied with the conversation in which you were given this bad news?

- o Yes, very satisfied
- o Yes, rather satisfied
- o Neither nor
- o No, rather dissatisfied
- o No, very dissatisfied

17. How long did the conversation last? ________ minutes

18. In which language did the conversation take place?

- German
- Arabic
- Turkish
- Russian
- Farsi
- Polish
- English
- French
- Spanish
- Other: _____________________

19. Did the conversation take place in the language you can best express yourself in?

- Yes, the medical staff/doctor spoke this language
- Yes, medical staff translated
- Yes, a relative translated
- Yes, a translator was present
- No

20. Were you able to understand the conversation at a linguistic level?

- Yes
- No

21. Were you offered to bring a relative for translation or even to organize a translator for the scheduled conversation?

- Yes
- No
- The conversation took place without prior appointment

22. Were you offered to bring a trusted person for support to the scheduled conversation?

- Yes
- No
- The conversation took place without prior appointment

23. Were you alone during the conversation, or did you have a trusted person with you?

- Yes, I brought someone with me
- Yes, someone happened to be there
- Yes, but I would have preferred to be alone
- No, but I would have preferred someone to be there with me
- No, I wanted to be alone during the conversation
- No, I was not aware of such an option

24. Did the conversation take place in a quiet setting?

- Yes
- Partly
- No

25. Did the medical staff take enough time for the conversation?

- Yes
- No

26. Did you have the feeling that the medical staff was under time pressure?

- Yes
- No

27. Where you overall satisfied with the general conditions during the conversation?

- Yes, very satisfied
- Yes, rather satisfied
- Neither nor
- No, rather dissatisfied
- No, very dissatisfied

28. Did the doctor meet you at your level of understanding concerning the medical part?

- Yes, I understood the medical part very well
- Yes, I understood the medical part rather well
- I was able to somewhat understand the technical part
- No, I had trouble understanding the medical part
- No, I was overwhelmed by the medical part

29. Did you feel overwhelmed by the amount of information during the conversation?

- Yes, very overwhelmed
- Yes, rather overwhelmed
- Neither nor
- No, rather not overwhelmed
- No, not overwhelmed at all

30. Were you able to remember the information shared with you during the conversation?

- Yes
- Partly
- No

31. Did you have enough time to ask questions?

- Yes
- Partly
- No

32. Did the medical staff adress your questions sufficiently?

- Yes
- Partly
- No

33. Were you offered a follow-up conversation?

- Yes, and I took advantage of it
- Yes, but I had no need
- No, and I had no need
- No, but I would have liked to have one

34. Who was the confidant that you first communicated the bad news to?

- Partner/Spouse
- Close friend
- Child(ren)
- Parent(s)
- Other family member(s)
- Psychologist
- Primary care physician/ general practitioner
- Outpatient gynecologist
- Other ________

35. When, after the conversation, were you able to talk about the bad news with your trusted person or persons?

- On the day of the conversation
- The day after the conversation
- Within a week after the conversation
- Within a month after the conversation
- A few months after the conversation
- Not at all
- Trusted person was present during the conversation

36. Did you feel overwhelmed when you communicated the bad news to your trusted person/your relatives?

- o Yes, very overwhelmed
- o Yes, rather overwhelmed
- o Partially
- o No, rather not overwhelmed
- o No, not at all overwhelmed

37. Would you have liked support when conveying the news to your relatives?

- o Yes
- o Partly
- o No

38. Can you please indicate your stress level after the conversation on a scale from 0 (not stressed at all) to 10 (extremely stresed).

Please circle the number on the thermometer below.

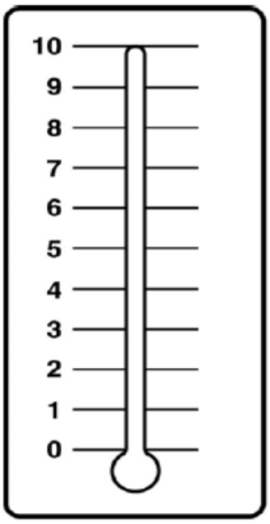

Extremely stressed

Not stressed at all

39. What has been a significant burden for you in this context? *(multiple answers possible)*

- o Change in body/physical changes
- o Physical weakness/fatigue
- o Changes in daily routine
- o Financial worries
- o Fear of losing autonomy
- o Fear of leaving family members alone
- o Fear of dying
- o Communicating the news to family members
- o Lack of support
- o Concerns regarding fertility/family planning
- o Uncertain planning

40. Were you also given any good news during the course of your illness?

- Yes
- No

41. Who did you communicate the good news to first?

- Partner/Spouse
- Close friend(s)
- Child(ren)
- Parent(s)
- Other family member(s)
- Psychologist
- Primary care physician/ general practitioner
- Outpatient gynecologist
- Other: ________________________________

42. Do you wish that the treating physician would give you good news more often?

- Yes
- No

43. Were you also informed of bad news through other means of communication?

- Yes, by phone
- Yes, by mail
- Yes, by e-mail
- Yes, other ____________________________
- No

44. What helped you the most most in dealing with the bad news?

Check all that apply.

- Support of family, partner, other relatives
- Support from doctor(s), treatment doctor, gynecologist, or primary care doctor
- Support from the treating psychologist
- Support groups for patients in similar conditions
- Being alone
- My faith
- Music
- Meditation
- Sports
- Art
- Literature
- Writing in a diary
- Hiking/being in nature
- Other: ____

45. How would you describe your current fear of receiving bad news?

- I feel better prepared for bad news.
- My fear of bad news remains unchanged.
- I currently have even greater fear of further bad news.

46. With which aspects and competencies of your doctor were you satisfied when receiving the bad news? *(multiple answers possible)*

- Professional competence
- Empathy
- Having conrete solutions
- Sufficient conversation time
- Taking my personal circumstances into consideration
- Encouragement to undergo therapy/motivational attitude
- None
- Other: ____

47. What did you miss from your doctor when receiving the bad news? *(multiple answers possible)*

- Professional competence
- Empathy
- Having concrete solutions
- Sufficient conversation time
- Taking my personal circumstances into consideration
- Encouragement to undergo therapy/motivational attitude
- Nothing
- Other: ____

48. Did you feel the doctor was well prepared for the conversation?

- Yes
- Partly
- No

If you answered *no* or *partly*: why did you have the feeling that the doctor was not well prepared?

- The doctor could not sufficiently answer my questions.
- The doctor was not adequately informed about my medical history.
- The doctor had to review the imaging after I entered the room.
- The doctor had to make a phone call to get further instructions.
- The doctor did not have any solutions ready.
- Other: ____

49. Were you satisfied with the non-verbal communication (e.g. body language, eye contact, tone) of the doctor?

- Yes, very satisfied
- Yes, rather satfisfied
- Neither nor
- No, rather dissatisfied
- No, very dissatisfied

50. How did the conversation change your relationship with the doctor?

- It greatly strengthened our relationship.
- It rather strengthened our relationship.
- The relationship remained unchanged.
- It rather weakened our relationship.
- It greatly weakened our relationship.

51. Do you think that doctors do not always tell the truth when delivering bad news?

- Yes
- No

52. How important is communication to you in general?

- Communication brings me joy.
- Communication makes me anxious.
- Communication bores me.
- Communication is an essential medium for me.
- I do not have a particular opinon on communication.

53. Which aspect is most important to you in your conversation partner?

Please rate each aspect on a scale from 1 to 10, where 1 means "not important at all" and 10 means "very important". Circle the number for each aspect.

Professional competence

1 2 3 4 5 6 7 8 9 10

Empathy

1 2 3 4 5 6 7 8 9 10

Appreciation

1 2 3 4 5 6 7 8 9 10

Honesty

1 2 3 4 5 6 7 8 9 10

54. Which information should definitely be communicated in such a conversation?

- o Nomal findingss
- o Changes in findings
- o Prognosis
- o Types of therapy
- o Side effects of the recommended therapies
- o Other: ____

55. How would you like to finish the conversation?

- o Specific information or instructions on the next steps
- o Fixed appointment for a follow-up conversation
- o I would prefer to call for a follow-up appointment when it suits me

56. In your opinion, what could have gone better in the conversation?

Check all that apply.

- o Communication of normal findings
- o More time for the conversation
- o Less technical terms
- o Speaking to medical staff who speak my native language
- o Having relatives with me who can translate
- o Offer to bring a trusted person for support
- o Having a professional translator present
- o Fewer interruptions during the session (phone, door or other distractions)

57. Were you satisfied with the amount of information you received about your illness and the further process at the end of the conversation?

- o Yes, very satisfied
- o Yes, rather satisfied
- o Neither nor
- o No, rather dissatisfied
- o No, very dissatisfied

58. Do you wish the doctor would provide you with informational material or suggest where you can get good information?

- o Yes, wish for brochures for patients
- o Yes, wish for suggestions for websites or apps
- o Yes, wish for tips for self-help groups
- o Yes, wish for informational material of any kind in my native language
- o No, not desired

59. Would you like to have more digital information sources?

- o Yes
- o No

60. Do you have any further suggestions for improvement?

____

____

____

____

____

____

- o No, no further suggestions for improvement.

61. How do you overall assess the need for improvement in delivering bad news?

- o There is much need for improvement.
- o There is a moderate need for improvement.
- o There is little need for improvement.
- o There is no need for improvement.
